# Molecular characterizations of genes in chloroplast genomes of the genus *Arachis* L. (Fabaceae) based on the codon usage divergence

**DOI:** 10.1371/journal.pone.0281843

**Published:** 2023-03-14

**Authors:** Shuwei Yang, Gun Li, Hao Li

**Affiliations:** 1 School of Intelligent Science and Information Engineering, Xi’an Peihua University, Xi’An, Shaanxi, China; 2 Department of Biomedical Engineering, Laboratory for Biodiversity Science, School of Electronic Information Engineering, Xi’An Technological University, Xi’An, Shaanxi, China; 3 College of Food Engineering, Jilin Engineering Normal University, Changchun, Jilin, China; Institute for Biological Research, University of Belgrade, SERBIA

## Abstract

Studies on the molecular characteristics of chloroplast genome are generally important for clarifying the evolutionary processes of plant species. The base composition, the effective number of codons, the relative synonymous codon usage, the codon bias index, and their correlation coefficients of a total of 41 genes in 21 chloroplast genomes of the genus *Arachis* were investigated to further perform the correspondence and clustering analyses, revealing significantly higher variations in genomes of wild species than those of the cultivated taxa. The codon usage patterns of all 41 genes in the genus *Arachis* were AT-rich, suggesting that the natural selection was the main factor affecting the evolutionary history of these genomes. Five genes (i.e., *ndhC*, *petD*, *atpF*, *rpl14*, and *rps11*) and five genes (i.e., *atpE*, *psbD*, *psaB*, *ycf2*, and *rps12*) showed higher and lower base usage divergences, respectively. This study provided novel insights into our understanding of the molecular evolution of chloroplast genomes in the genus *Arachis*.

## Introduction

As one of the most important economical crops, peanut (*Arachis hypogaea* L.) is an annual crop in the legume family (Fabaceae), cultivated for edible oil and food in more than 100 countries worldwide. To date, a large number of germplasm resources of the genus *Arachis* are maintained in China, India, and the United States. It is well known that genetic diversity declines in proportion to the severity of the genetic bottleneck. Due to the significant genetic bottleneck in the cultivated taxa of peanuts, these plants generally show a narrow genetic base [1]. Although the cultivated taxa of peanuts are classified via morphological characters (i.e., the presence or absence of axles on the main stem), the genetic variation is generally considered the fundamental element of species diversity, it is necessary to study the genetic divergence of the genus *Arachis* due to its agricultural significance [2,3]. Advances in novel genomic tools are helpful for illuminating the evolution of cultivated and wild taxa of the genus *Arachis* [4], while the genomic information is important for studying the molecular characteristics of peanuts based on selected genes.

The genetic codons are closely linked to nucleic acids and proteins. Therefore, the codon usage patterns of a selected gene are important for exploring its molecular functions [5], i.e., predicting its degree of inheritance as well as its adaptiveness during evolution. Studies have investigated the significance of the molecular composition and the codon usage pattern at the genomic and genetic levels based on chloroplast genomes for exploring the genetic diversity within plants [6,7]. For example, studies have revealed that the codon usage patterns in chloroplast genomes reflect the degrees of genetic variations under the evolutionary pressure [8,9]. The chloroplast genomes contain molecular characteristics important for both clarification of the evolutionary history and improvement of crop plants. Therefore, comparative analyses based on codon usage patterns of chloroplast genomes have been widely used to evaluate the genetic correlation among groups of plants [10,11]. Further, the relationship among the compositions, such as the relationship between GC12 and GC3 of chloroplast genomes could be also used to distinguish the sub-genus of a plant [12].

Peanuts provide a large portion of nutrients for human populations in China, India, and many countries in South Saharan Africa [13]. Peanut is an important source of high-quality cooking oil and is also appreciated worldwide as a type of affordable and flavorful food [14]. For a long term, peanuts have been used either as a whole or as an ingredient in food to provide the highest protein contents among many commonly consumed snack nuts, and served as a rich source of heart-healthy, monounsaturated lipids [15]. Studies have shown that molecular characteristics of plant genomes are generally influenced by many human or natural factors [16]. Furthermore, plant biodiversity is important for the ecological investigations and is determined by many factors, such as the geographical locations [17,18], genome and gene structures [19,20], and the temporal factors [21]. A large number of studies have provided a solid foundation for understanding not only the chemical compositions of peanuts, but also the breeding methods to improve the quality of peanuts. For example, the plant chloroplast genomes are important for photosynthesis and have been usually used as the molecular systems to investigate the gene expressions [22]. Furthermore, the codon usage patterns in plant genomes have been used as the evolutionary characteristics to perform phylogenetic analysis. For example, studies have explored the genomic evolution and phylogenetic development in plant chloroplasts based on the variations in their compositions and the maximum likelihood of sequences [23–26]. Moreover, the molecular and genetic analyses of the chloroplast genomes have provided solid experimental evidence to facilitate the improvements in crop plants [27,28].

The chloroplast genome is composed of a single circular double stranded DNA molecule, capable of independent replication and transcription. Compared with the nuclear genome, the chloroplast genome shows unique characteristics in its base ratio, nucleotide sequence, and gene structure. Many factors, such as the environment and the cultivation by humans, affect the evolutionary characteristics of the chloroplast genome. Due to their relatively small sizes, it is generally cost effective and convenient to obtain and analyze the chloroplast genomes compared to the nuclear genomes [29]. However, studies on the diversity degree of both genes and the overall molecular characterizations of chloroplast genomes in the genus *Arachis* are sparse [30]. Despite the well-established taxonomic groupings of the genus *Arachis*, the evolutionary characteristics and genetic diversity of chloroplast genomes in the genus *Arachis* are not clear due to their relatively conserved genomes and lack of appropriate data. For example, the codon usage bias and divergences of genes in chloroplast genomes of the genus *Arachis* would be imperative data for studying their variations of molecular adaptation as well as their molecular diversities.

As the main food production systems, crops play an important role in nutritional security. With the rapidly increasing population worldwide, there is an urgent need to increase the crop production in order to ensure the food security in the near future [31]. Molecular studies enhanced by the next generation DNA sequencing technology allow the extensive exploration of the structural and functional features of plant genomes, which are expected to show significant impact on not only the fundamental biological studies of plants but also the genetic improvement of crops [32]. For example, comprehensive studies on the codon usage patterns of a plant could reveal the key factors in codon choice and its molecular evolution [33]. It is well known that the genes in chloroplast genomes are generally highly conserved, with some of them commonly used as the molecular markers for taxonomic identification [34]. In the present study, the codon usage divergences and their potential taxonomic applications were explicitly investigated based on a total of 21 chloroplast genomes of the genus *Arachis* available at the National Center for Biotechnology Information (NCBI, https://www.ncbi.nlm.nih.gov/) database. The codon usage patterns of these genomes were explored based on several molecular parameters, including the basic composition, the effective number of codons (ENCs), the codon bias index (CBI), and the relative synonymous codon usage (RSCU) of a total of 41 genes shared in these chloroplast genomes. The codon usage variations among these genes were further assessed to evaluate the factors affecting the evolutionary history of these chloroplast genomes. Genes with varied codon usage divergence and base usage divergence were identified. This study provided novel evidence to support the further investigations of the molecular evolution of chloroplast genomes of *Arachis* species.

## Materials & methods

### Selection of chloroplast genomes and genes

A total of 21 completely sequenced chloroplast genomes of 14 species of the genus *Arachis*, including *A*. *batizocoi*, *A*. *cardenasii*, *A*. *correntina*, *A*. *diogoi*, *A*. *duranensis*, *A*. helodes, *A*. *hoehnei*, *A*. *ipaensis*, *A*. *paraguariensis*, *A*. *pintoi*, *A*. *stenosperma*, *A*. *villosa*, *A*. *hypogaea* (with 8 accessions), and *A*. *monticola*, were retrieved in the NCBI database. A total of 41 genes shared in all 21 chloroplast genomes (i.e., *accD*, *atpA*, *atpB*, *atpE*, *atpF*, *atpI*, *ccsA*, *cemA*, *clpP*, *matK*, *ndhA*, *ndhC*, *ndhE*, *ndhF*, *ndhG*, *ndhH*, *ndhJ*, *petA*, *petD*, *psaA*, *psaB*, *psbA*, *psbB*, *psbD*, *rbcL*, *rpl14*, *rpl20*, *rpoA*, *rpoB*, *rpoC1*, *rps2*, *rps3*, *rps4*, *rps7*, *rps8*, *rps11*, *rps12*, *rps14*, *ycf2*, *ycf3*, and *ycf4*) were selected for further analyses. To facilitate the analyses performed in this study, the selection of these genes was based on the following criteria: (1) the gene sequences were longer than 300 bp, (2) the starting codon of these genes was ATG, and (3) the total number of the bases was divisible by 3. Further, for the standardization of genes in different genomes, some genes with total sequence quantity less than 21 or 42 (for those double copy genes) were excluded.

### Statistics of the molecular characteristics in gene sequences

In order to investigate the codon usage patterns, the basic molecular components of the gene sequences, including the occurrences of the adenine (A), the thymine (T), the cytosine (C), and the guanine (G), the contents of the third bases, the GC12, the GC3, as well as the overall GC contents and the number of codons were calculated based on the Matlab 2010b platform using the in-house scripts.

### Calculation of the codon usage pattern

The effective numbers of codon (ENC) values of 41 genes in the genus *Arachis* were used to quantify their degrees of codon usage bias. The lower ENC value indicated that the inner codon usage was more biased, with 35 generally regarded as the bias threshold of the codon usage pattern [35]. Based on the codon quantity of the gene sequences counted, the ENC values of each gene were calculated by using the following Eq (1) [36,37]:

$$ENC^{\mathrm{calculated}}=2+\frac{9}{{\bar{f}}_{2}}+\frac{1}{{\bar{f}}_{3}}+\frac{5}{{\bar{f}}_{4}}+\frac{3}{{\bar{f}}_{6}}$$ (1)

where ${\bar{f}}_{k}$ (*k* = 2, 3, 4, and 6) was the mean of *f*_*k*_ values. The *f*_*k*_ value was calculated by the following Formula (2) for the *k*-fold degenerate amino acids:

$$f_{k}=\frac{n\sum_{i=1}^{k}{\left(n_{i}/n\right)}^{2}-1}{n-1}$$ (2)

where *n* was the total number of occurrences of the codons for that amino acid, and the *n*_*i*_ was the total number of occurrences of the *i*-th codon for that amino acid. The relationship between the ENC values and the GC3s ratios was generally used to evaluate the homogeneous property of codon usage in the genes. The comparison between the ENC values with the expected value calculated by ENC^*expected*^ = 2+s+{29/[s^2^+(1-s)^2^]} [38], with the parameter *s* representing the given composition of GC3s, was used to evaluate the evolutionary pressure in the genes.

The bias of base content within each gene of the chloroplast genomes of the genus *Arachis* was evaluated by the PR2 plot, with the AT-bias [A/(A + T)] plotted as the Y-axis and the GC-bias [G/(G + C)] as the X-axis [39]. The neutrality plot with the scatter diagram based on GC12 against GC3 of the gene sequences was used to identify the factors, i.e., the mutation pressure and the natural selection during the evolutionary history, influencing the evolutionary pressure in the genes. The relative synonymous codon usage (RSCU) values of genes of the genus *Arachis* in chloroplast genomes were calculated by the following Formula (3) [40]:

$$RSCU=\frac{g_{ij}}{\sum_{j}^{n_{i}}g_{ij}}\cdot n_{i}$$ (3)

where the parameter *g*_*ij*_ denoted the observed number of the *i*-th codon for the *j*-th amino acid, and the *n*_*i*_ represented the quantity of the types of synonymous codons for the amino acid. The RSCU values of genomes have been generally used for evaluating the bias of the synonymous codons [41]. The ideal RSCU value of a codon is equal to 1 if there is only the mutation affecting the codon usage pattern [42]. The higher RSCU value indicates that the corresponding codon is used more frequently in the gene. The codon is defined as more-abundant with its RSCU value larger than 1.0, suggesting that the codon is favored over the other codons, whereas the codon is considered as less-abundant with its RSCU value less than 1.0 [43,44].

As commonly used to describe the foreign gene expression in the host, the codon bias index (CBI) of the genes in the chloroplast genomes of the genus *Arachis* was calculated by the following Formula (4) [45]:

$$CBI=\frac{N_{opt}-N_{ran}}{N_{tot}-N_{ran}}$$ (4)

where the *N*_*opt*_ represented the total number of codon appeared in the superior sequences, the *N*_*ran*_ represented the sum of codons for the occurrences of the superior codon when all the synonymous codons were randomly distributed, and the *N*_*tot*_ indicated the number of occurrence of the amino acid corresponding to the superior codon in the genes.

### Codon usage divergence analysis

The protein length *vs*. GC ratio of each gene sequence was calculated to explore the influence of the sequence length on the GC ratio. Similarly, the influence of the ENC on the CBI in all gene sequences was assessed to study the codon usage pattern of all 41 genes. The correspondence analysis and the clustering analyses were further performed to investigate the evolutionary distances among the 21 chloroplast genomes of the genus *Arachis* based on their RSCU values. The divergences of codon usage in all 41 gene sequences were calculated by summing the standard deviations of all their codon usage parameters.

## Results

### Base usage of chloroplast genes in the genus *Arachis*

**Fig 1:**
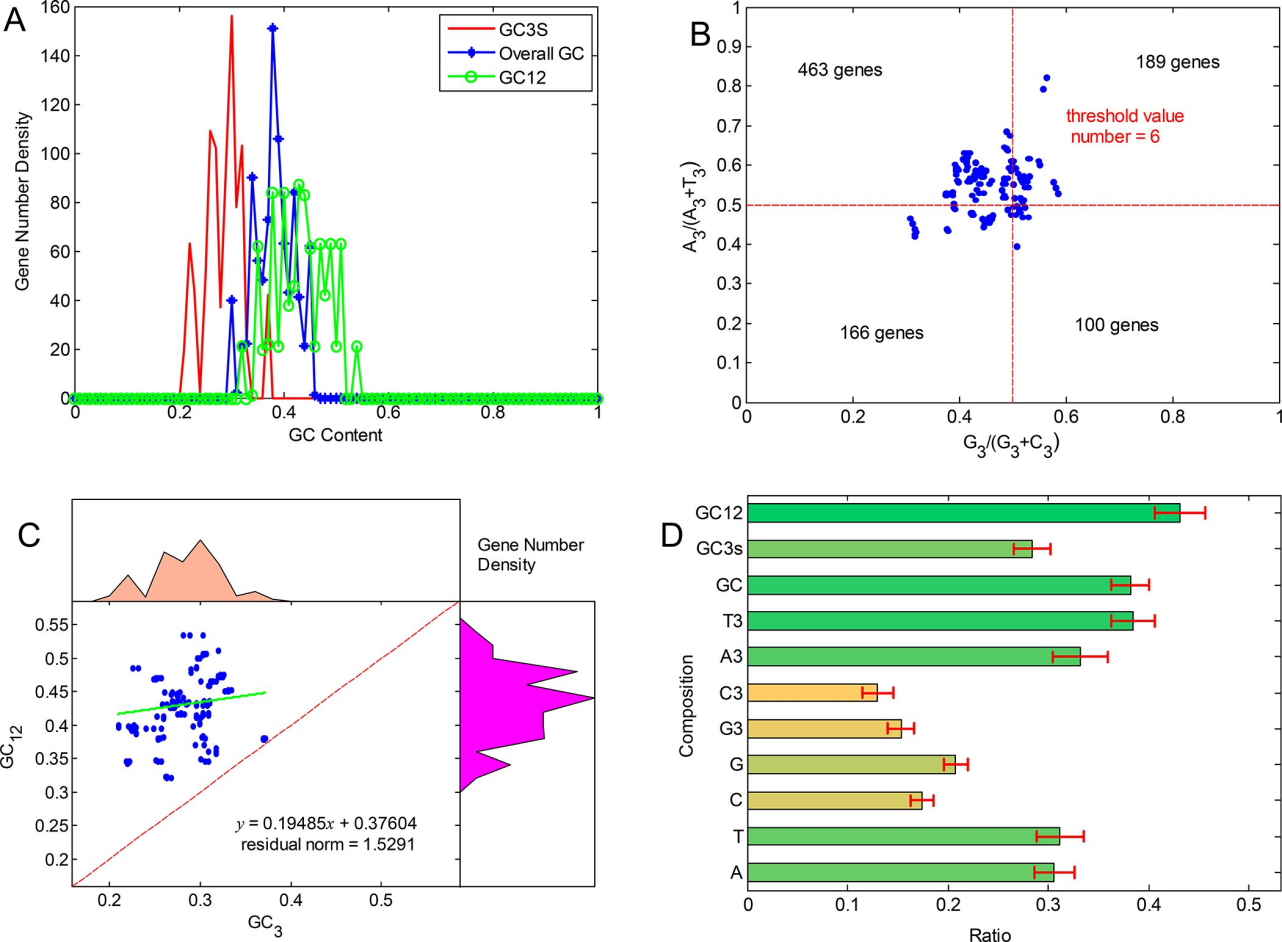
Molecular characterizations of the 41 genes in 21 chloroplast genomes of the genus *Arachis*. (**A**) Distributions of overall GC, GC12, and GC3s contents. (**B**) The PR2 plot. (**C**) The neutrality plot. (**D**) The base ratios of overall 41 genes in 21 chloroplast genomes.

The contents of GC12, GC3s, and the overall GC of the 924 gene sequences (S1 Table in S1 File), including the 41 genes (3 with two copies) in 21 chloroplast genomes of the genus *Arachis*, were shown in the area graph (Fig 1A). The results showed that all 3 types of GC contents of these genes were less than 50%, revealing evidently that these gene sequences were AT-biased. The results of the PR2 bias plot with G_3_/(G_3_+C_3_) as X-axis and A_3_/(A_3_+T_3_) as Y-axis for these gene sequences showed no evident bias within the usage of the third bases of the codons (Fig 1B). The results of the neutrality plot based on the relationship between GC_12_/(GC_12_+AT_12_) and GC_3_/(GC_3_+AT_3_) for all gene sequences revealed that the ratios of GC3 ranged largely from 20% to 35%, while the ratios of GC12 mainly ranged from 35% to 50%, with both roughly showing normally distributed patterns (Fig 1C). The compositions for each of the three positions in codons were calculated to explore the overall base usage (Fig 1D). The results showed that the compositions of A and T varied over a larger range than that of the compositions of G and C at both the 3-base level and the third base level.

### Codon usage within chloroplast genes of the genus *Arachis*

**Fig 2:**
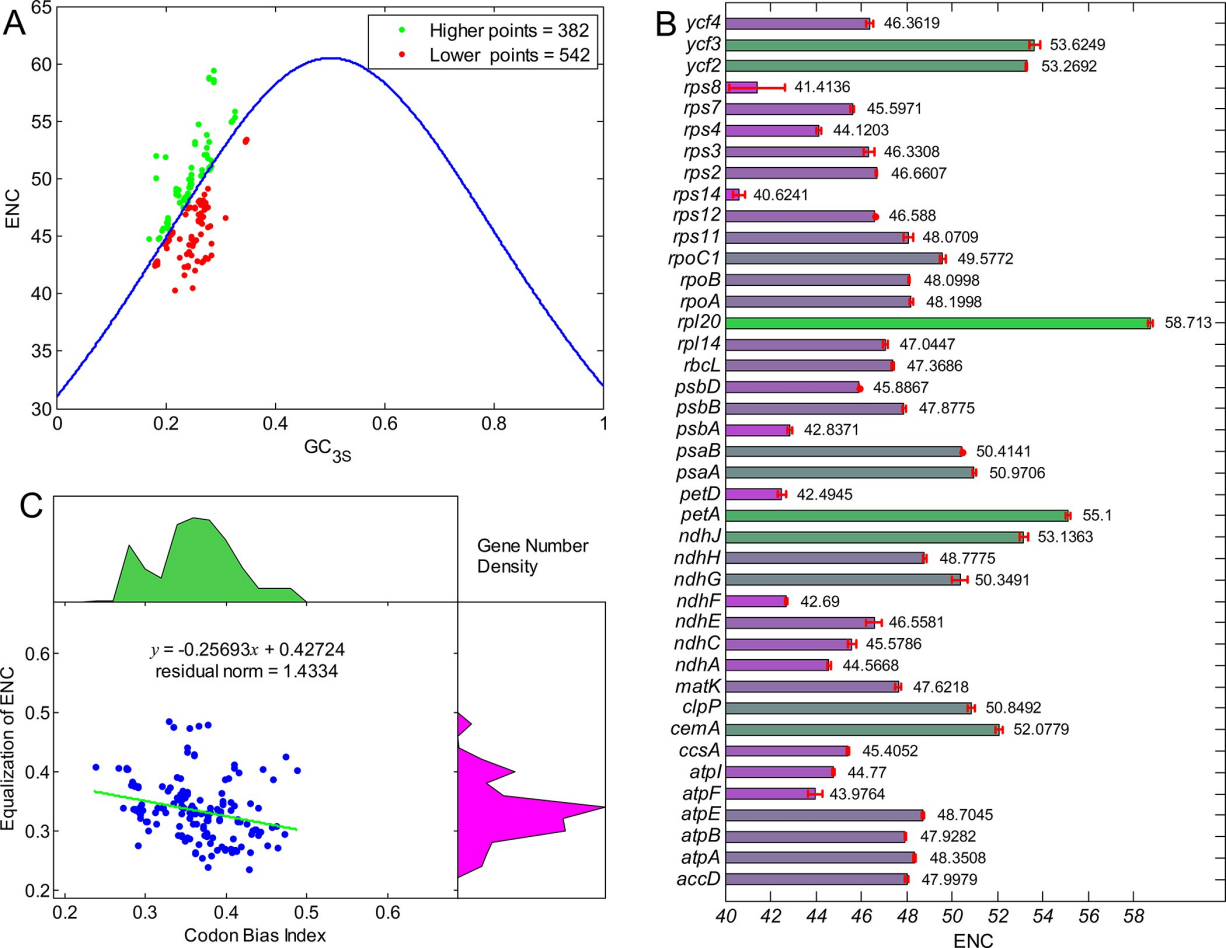
Codon usage patterns in chloroplast genes of the genus *Arachis*. (**A**) Effective number of codon (ENC) *vs*. GC3s plot. The blue curve denotes the expected theoretical ENC values with no evolutionary constraint, the dots under the blue line indicate bias in the genes, and the dots above the line denote little or no bias but homogeneous codon usage in these gene sequences. (**B**) The ENC values for individual genes. (**C**) Relationships between the ENC values (normalized to the range of the CBI distributions) and the CBI values of 41 genes in 21 chloroplast genomes of the genus *Arachis*.

As usually used to evaluate the evolutionary pressure of a gene, the ENC values of the 41 genes in 21 chloroplast genomes of the genus *Arachis* were calculated and presented by the ENC-GC3s plot (Fig 2A). The results showed that most of the data points were below the standard curve, suggesting that the corresponding genes were more biased and under both evolutionary pressures (i.e., natural selection pressure and mutation pressure). The ENC values were also displayed separately for these genes (Fig 2B). The results showed that the standard deviations for different genes were significantly different, revealing that the ENC values for certain genes (i.e., the *rps8*, *ndhG*, and *atpF*) were variable. The ENC values were further normalized to the range of the CBI values, with the fitting results showing evidently the negative correlations between the codon bias and the ENC values, as indicated by the correlation coefficient of -0.26 (Fig 2C).

**Fig 3:**
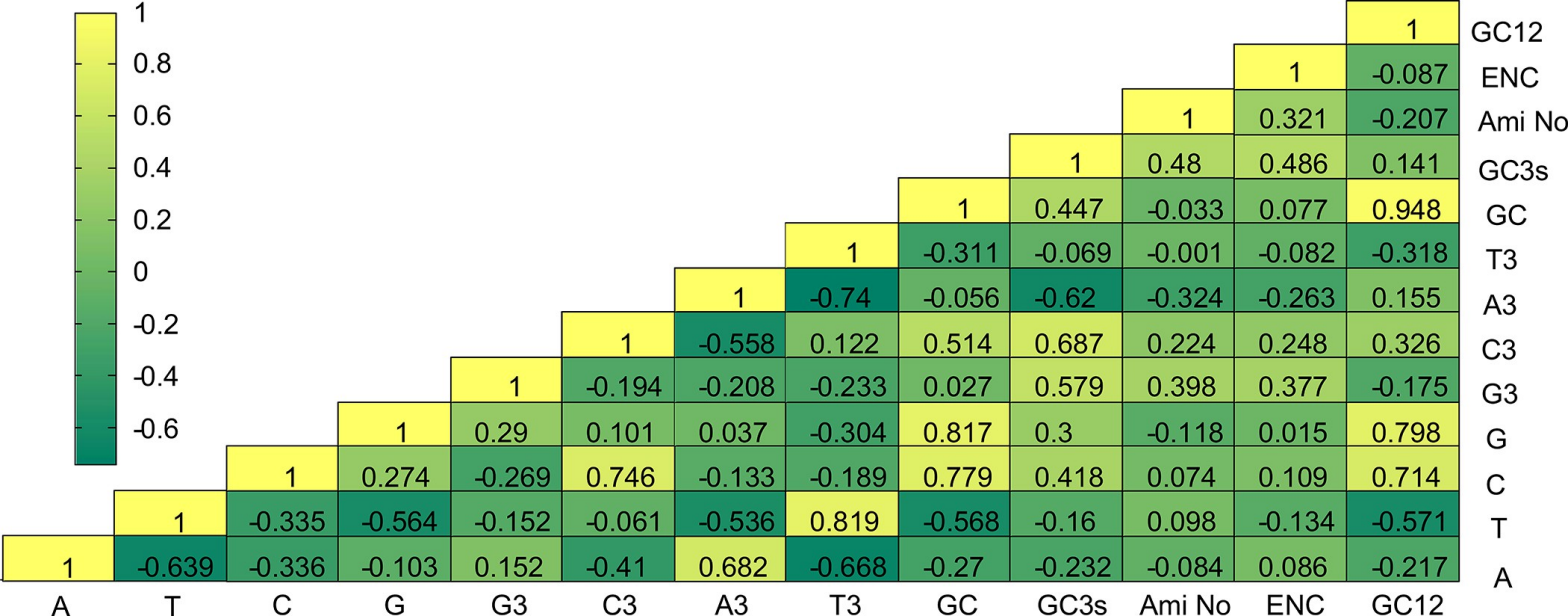
Correlation coefficients of the correlation analyses among the codon usage parameters of the 41 genes in 21 chloroplast genomes of genus *Arachis*. “Ami No” represents the total number of amino acids based on the gene.

The correlation analysis was performed to identify the relationships among the codon usage parameters, i.e., the ENC, the compositions of G, C, A, and U, and the length of protein encoded by genes, of the 41 genes in 21 chloroplast genomes of the genus *Arachis* (Fig 3). The results revealed higher GC3s rate (correlation coefficient = 0.480) and higher ENC values (correlation coefficient = 0.321) in the longer genes, while the ENC values showed positive correlation with the GC3s (correlation coefficient = 0.486).

**Fig 4:**
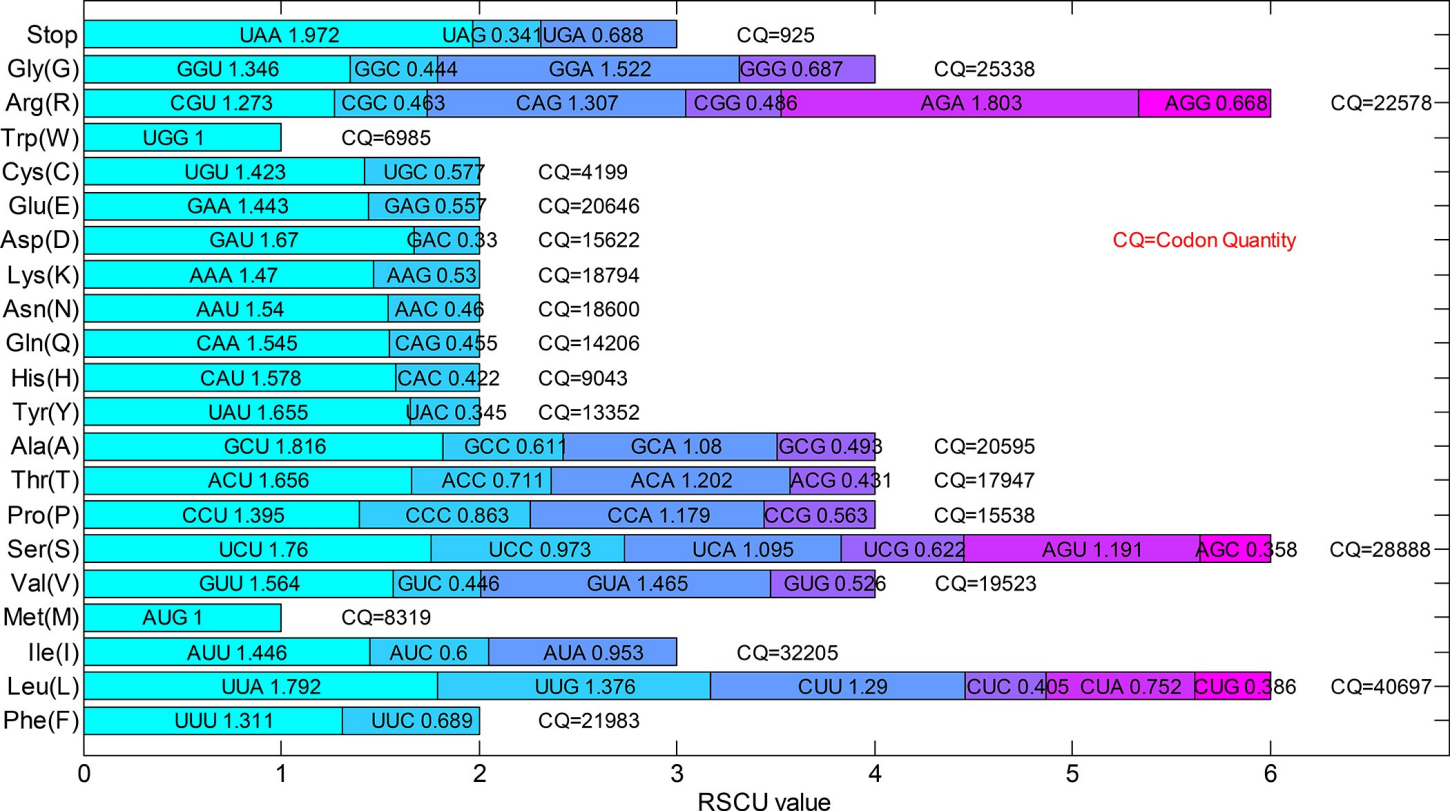
The relative synonymous codon usage (RSCU) values of 41 genes in 21 chloroplast genomes of the genus *Arachis*. The names of amino acids are abbreviated and listed along the Y-axis. “Stop” represents the terminal codons of UAA, UAG, and UGA. The CQ values represent the total codons in the 924 coding sequences.

To reveal the proportion of codons encoding the identical amino acid in the 41 genes of 21 chloroplast genomes of the genus *Arachis*, the RSCU values and the codon quantity of these genes, including the three terminal codons (UAA, UAG, and UGA) and the one-dimensional degenerate codons (AUG and UGG), were calculated (Fig 4). The results showed that the abundant codons with RSCU values higher than 1.5 included UUA, GUU, UCU, ACU, GCU, UAU, CAU, CAA, AAU, GAU, AGA, and GGA, while the less-abundant codons with RSCU values less than 0.5 included CUC, CUG, AGC, ACG, GCG, UAC,CAC, CAG, AAC, GAC, CGC, AGG, and GGC. It was noted that about two-thirds of the terminal codons were UAA.

### Codon usage divergences of chloroplast genes of the genus *Arachis*

**Fig 5:**
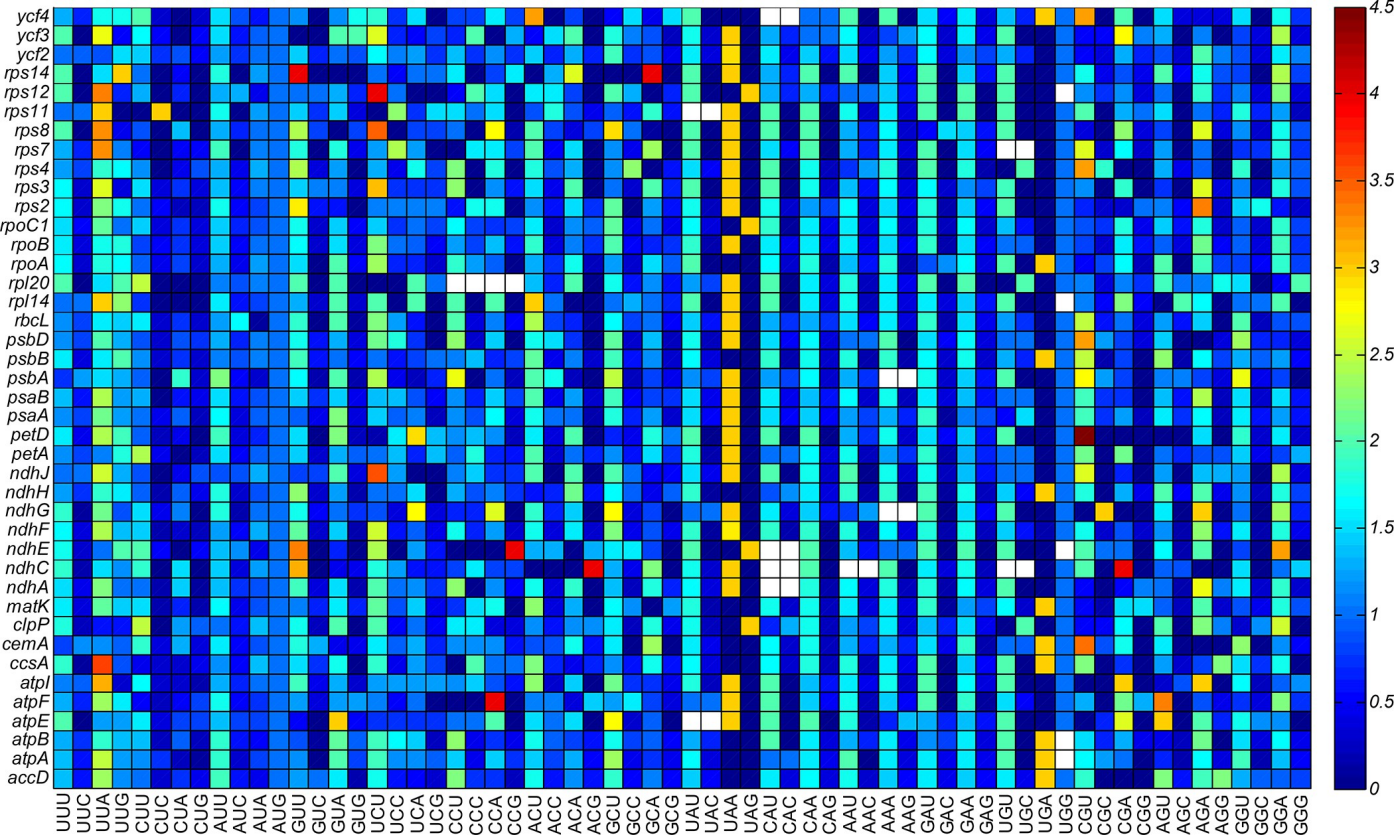
Heat map of the relative synonymous codon usage (RSCU) values for the 41 genes in 21 chloroplast genomes of the genus *Arachis*. The blank boxes indicate the absence of corresponding codons for certain amino acids.

It was noted that the codon usage divergences of individual genes could not be evaluated appropriately by the overall codon usage patterns (Figs 1–4). Therefore, the RSCU values for the 41 genes in 21 chloroplast genomes of the genus *Arachis* were calculated separately (Fig 5). The distributions of RSCU values varied greatly among these genes, showing that the RSCUs among the chloroplast genomes of the genus *Arachis* were different from each other as detected by the independent considerations of these genes, while the codon usage preferences in these genes were of significant differences. The results also showed that not all genetic codons were detected for some amino acids. For example, no codons for tryptophan (Trp) were revealed in the genes *atpA*, *atpB*, *ndhE*, *rpl1*, and *rps1* in all 21 chloroplast genomes. Similarly, no corresponding codons were found for histidine (His), asparagine (Asn), and cystine (Cys) in gene *ndhC* of all 21 chloroplast genomes.

**Fig 6:**
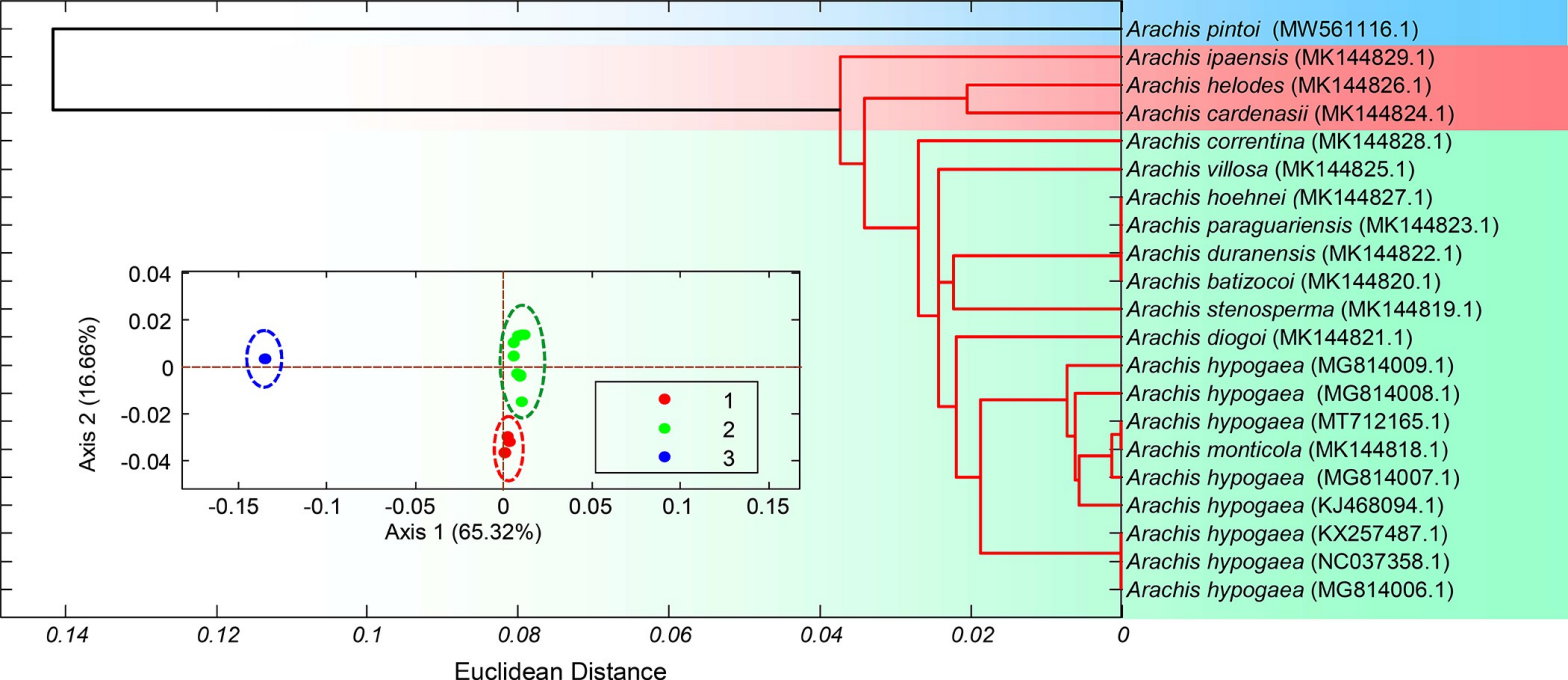
Variations among 21 chloroplast genomes of the genus *Arachis* based on the Euclidean distances calculated by the RSCU values of 41 genes in each chloroplast genome and the correspondence analysis (presented in the inset graph) of 21 chloroplast genomes of the genus *Arachis*.

The variations of the RSCU values of the 41 genes among the 21 chloroplast genomes of the genus *Arachis* were evaluated by their Euclidean distances to reveal the differences on the relative codon usage for certain amino acids (Fig 6). The correspondence analysis of these chloroplast genomes was conducted based on their RSCU values with the exclusions of three terminal codons and the codons for Met and Trp (the inset graph of Fig 6). Both analyses revealed largely congruent groupings among the 21 chloroplast genomes of the genus *Arachis*, which were realized into three groups with the chloroplast genome of *A*. *pintoi* recognized in one group, three species (i.e., *A*. *ipaensis*, *A*. *helodes*, and *A*. *cardenasii*) revealed in another group, and the other 17 chloroplast genomes in the third group. All eight accessions of *A*. *hypogaea* were revealed in one clade with the inclusion of *A*. *monticola* as well. The Euclidean distances among the RSCU values revealed significantly higher variations in genomes of wild species of *Arachis* than those of the cultivated taxa (i.e, *A*. *hypogaea*).

The codon usage divergences of the 41 genes in 21 chloroplast genomes of the genus *Arachis* were also evaluated by their base usage diversity. In order to further explore the divergences of different genes at the base usage level, the 41 genes in 21 chloroplast genomes of the genus *Arachis* were considered simultaneously to calculate their base usage (Table 1). The GC3 contents for all genes were lower than 50%, while the GC12 contents in some genes (i.e., *psbB*, *rbcL*, and *rps11*) were higher than 50%. These results showed that although the GC content for all genes could be plotted (Fig 1A), it is still necessary to explicitly identify the GC contents for individual genes. Based on the standard deviations, the GC12 content for all 41 genes were more consistent than that of the GC3.

**Table 1:** Base usage diversity of 41 genes in 21 chloroplast genomes of the genus *Arachis*. Data are presented as mean ± standard deviation. Two copies of each of the three genes (i.e., *rps12*, *rps7*, and *ycf2*) are detected in each of the 21 chloroplast genomes.

Gene (number)	A	T	C	G	GC	GC3	GC12
*accD* (n = 21)	0.3251±0.0005	0.3180±0.0004	0.1488±0.0004	0.2078±0.0003	0.3568±0.0004	0.3020±0.0004	0.3836±0.0008
*atpA* (n = 21)	0.3139±0.0004	0.2880±0.0002	0.1790±0.0002	0.2181±0.0005	0.3973±0.0008	0.2504±0.0014	0.4711±0.0004
*atpB* (n = 21)	0.3010±0.0000	0.2750±0.0002	0.1890±0.0002	0.2339±0.0004	0.4239±0.0002	0.2950±0.0000	0.4879±0.0002
*atpE* (n = 21)	0.3458±0.0006	0.2670±0.0000	0.1701±0.0006	0.2170±0.0000	0.3881±0.0004	0.2740±0.0000	0.4441±0.0008
*atpF* (n = 21)	0.3497±0.0004	0.2828±0.0027	0.1477±0.0018	0.2204±0.0009	0.3679±0.0032	0.2967±0.0037	0.4031±0.0027
*atpI* (n = 21)	0.2689±0.0004	0.3615±0.0009	0.1872±0.0004	0.1830±0.0005	0.3702±0.0006	0.2749±0.0021	0.4170±0.0004
*ccsA* (n = 21)	0.2900±0.0004	0.3930±0.0002	0.1425±0.0010	0.1743±0.0007	0.3168±0.0003	0.2554±0.0015	0.3470±0.0004
*cemA* (n = 21)	0.3188±0.0006	0.3463±0.0011	0.1858±0.0006	0.1490±0.0004	0.3348±0.0006	0.3083±0.0021	0.3480±0.0004
*clpP* (n = 21)	0.3006±0.0008	0.2795±0.0012	0.1858±0.0006	0.2340±0.0000	0.4198±0.0008	0.2890±0.0000	0.4847±0.0010
*matK* (n = 21)	0.3162±0.0007	0.3799±0.0004	0.1521±0.0005	0.1510±0.0005	0.3031±0.0004	0.2621±0.0014	0.3245±0.0006
*ndhA* (n = 21)	0.2820±0.0000	0.3770±0.0004	0.1640±0.0003	0.1769±0.0002	0.3410±0.0004	0.2248±0.0013	0.3981±0.0006
*ndhC* (n = 21)	0.2405±0.0012	0.4117±0.0017	0.1411±0.0016	0.2064±0.0015	0.3476±0.0027	0.2494±0.0080	0.3970±0.0000
*ndhE* (n = 21)	0.2710±0.0000	0.3811±0.0013	0.1641±0.0018	0.1830±0.0000	0.3471±0.0018	0.2968±0.0046	0.3730±0.0000
*ndhF* (n = 21)	0.2919±0.0003	0.4036±0.0007	0.1460±0.0003	0.1584±0.0005	0.3043±0.0007	0.2203±0.0012	0.3464±0.0007
*ndhG* (n = 21)	0.2579±0.0004	0.4050±0.0010	0.1678±0.0006	0.1691±0.0008	0.3370±0.0014	0.2098±0.0037	0.4007±0.0009
*ndhH* (n = 21)	0.3148±0.0006	0.3050±0.0007	0.1460±0.0002	0.2340±0.0002	0.3810±0.0004	0.2717±0.0013	0.4351±0.0009
*ndhJ* (n = 21)	0.3020±0.0000	0.3118±0.0006	0.1740±0.0000	0.2121±0.0006	0.3861±0.0006	0.2962±0.0013	0.4311±0.0006
*petA* (n = 21)	0.3021±0.0006	0.2867±0.0008	0.1900±0.0003	0.2210±0.0006	0.4110±0.0007	0.3298±0.0014	0.4521±0.0003
*petD* (n = 21)	0.2751±0.0007	0.3363±0.0009	0.1872±0.0021	0.2002±0.0019	0.3876±0.0009	0.2700±0.0048	0.4464±0.0014
*psaA* (n = 21)	0.2561±0.0004	0.3207±0.0004	0.2039±0.0003	0.2191±0.0003	0.4230±0.0003	0.3208±0.0012	0.4743±0.0006
*psaB* (n = 21)	0.2560±0.0000	0.3340±0.0000	0.1919±0.0002	0.2190±0.0000	0.4100±0.0000	0.3099±0.0002	0.4610±0.0000
*psbA* (n = 21)	0.2340±0.0002	0.3493±0.0009	0.1939±0.0006	0.2216±0.0007	0.4156±0.0011	0.3128±0.0034	0.4680±0.0000
*psbB* (n = 21)	0.2340±0.0000	0.3256±0.0005	0.1809±0.0002	0.2594±0.0005	0.4403±0.0006	0.3052±0.0008	0.5079±0.0002
*psbD* (n = 21)	0.2210±0.0000	0.3530±0.0002	0.2039±0.0002	0.2210±0.0000	0.4259±0.0002	0.3248±0.0006	0.4760±0.0000
*rbcL* (n = 21)	0.2720±0.0000	0.2940±0.0002	0.1899±0.0002	0.2431±0.0004	0.4331±0.0003	0.2962±0.0007	0.5020±0.0000
*rpl14* (n = 21)	0.3327±0.0007	0.2787±0.0010	0.1651±0.0006	0.2225±0.0012	0.3885±0.0012	0.2619±0.0043	0.4510±0.0000
*rpl20* (n = 21)	0.3812±0.0010	0.2720±0.0000	0.1362±0.0009	0.2103±0.0014	0.3465±0.0014	0.3157±0.0032	0.3629±0.0014
*rpoA* (n = 21)	0.3661±0.0003	0.2934±0.0010	0.1603±0.0014	0.1801±0.0003	0.3404±0.0011	0.2571±0.0019	0.3825±0.0009
*rpoB* (n = 21)	0.3250±0.0002	0.2907±0.0005	0.1660±000000	0.2183±0.0004	0.3842±0.0005	0.2857±0.0007	0.4341±0.0005
*rpoC1* (n = 21)	0.3268±0.0014	0.2982±0.0015	0.1646±0.0005	0.2102±0.0005	0.3749±0.0003	0.2681±0.0008	0.4282±0.0005
*rps11* (n = 21)	0.3051±0.0004	0.2423±0.0021	0.2083±0.0014	0.2451±0.0004	0.4527±0.0014	0.2870±0.0045	0.5360±0.0000
*rps12* (n = 42)	0.3030±0.0000	0.2490±0.0000	0.2270±0.0000	0.2210±0.0000	0.4480±0.0000	0.3190±0.0000	0.5130±0.0000
*rps14* (n = 21)	0.3528±0.0006	0.2640±0.0000	0.1521±0.0006	0.2310±0.0000	0.3831±0.0006	0.2774±0.0021	0.4360±0.0000
*rps2* (n = 21)	0.3328±0.0004	0.2929±0.0004	0.1659±0.0002	0.2085±0.0009	0.3744±0.0008	0.2952±0.0010	0.4143±0.0008
*rps3* (n = 21)	0.3900±0.0002	0.2740±000000	0.1396±0.0007	0.1964±0.0009	0.3359±0.0002	0.2278±0.0008	0.3900±0.0008
*rps4* (n = 21)	0.3401±0.0006	0.2840±0.0003	0.1947±0.0007	0.1819±0.0004	0.3757±0.0007	0.2612±0.0017	0.4329±0.0004
*rps7* (n = 42)	0.3569±0.0004	0.2438±0.0006	0.1861±0.0004	0.2141±0.0004	0.4001±0.0005	0.2246±0.0020	0.4870±0.0000
*rps8* (n = 21)	0.3557±0.0009	0.2838±0.0010	0.1461±0.0010	0.2151±0.0004	0.3604±0.0010	0.2533±0.0028	0.4148±0.0008
*ycf2* (n = 42)	0.3109±0.0002	0.3120±0.0002	0.1840±0.0000	0.1930±0.0002	0.3770±0.0002	0.3699±0.0004	0.3809±0.0002
*ycf3* (n = 21)	0.3206±0.0008	0.2862±0.0007	0.1870±0.0006	0.2061±0.0014	0.3931±0.0010	0.3052±0.0024	0.4375±0.0016
*ycf4* (n = 21)	0.2575±0.0008	0.3639±0.0004	0.1514±0.0012	0.2270±0.0007	0.3785±0.0011	0.3034±0.0015	0.4165±0.0012

**Fig 7:**
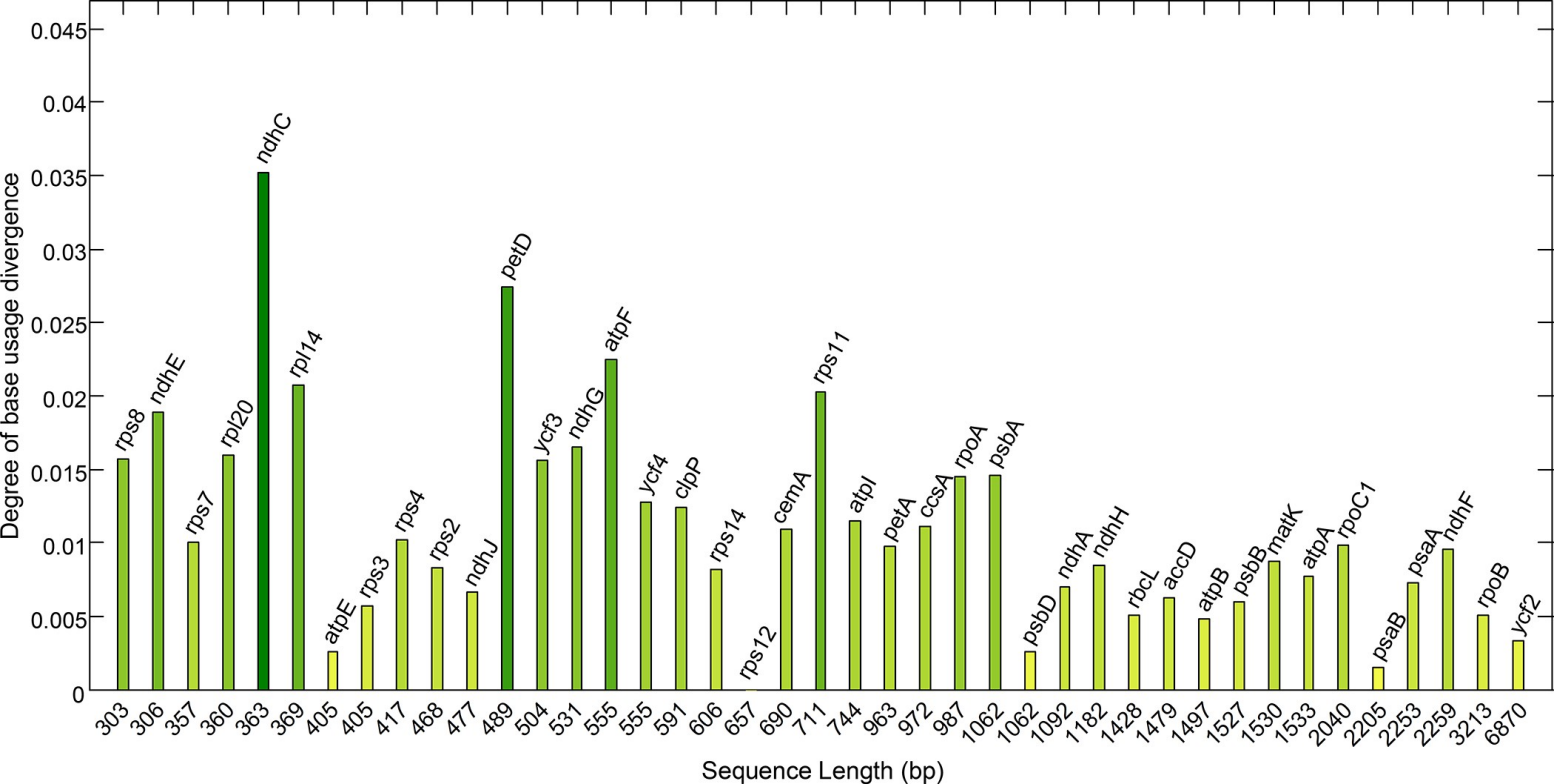
Degree of base usage divergences of 41 genes in 21 chloroplast genomes of the genus *Arachis*. The X-axis represents the length of gene sequences.

To explore the degree of the base usage diversity in the 41 genes, the standard deviation values of base component parameters, including percentages of A, G, C, T, G3, C3, A3, T3, GC12, GC3, and overall GC of each gene were calculated and summed (Fig 7). The results showed that the diversities of these genes were not only affected by the mutations in the genes, but also by the sequence lengths of these genes. The mutations in shorter genes showed evidently larger impact on the composition than that in the longer genes, while some longer genes, such as *ycf2*, *psbB*, *atpE* and *psbD*, were generally of lower base usage diversity. Among the 41 genes, the *rps12* contained the most stable sequence (base usage divergence = 0) showing no variations in their sequences of 21 chloroplast genomes. Further, double-copy genes showed relatively more stable characteristics, such as the genes with longer sequences *ycf2* and *rps7*, and the gene with shorter sequence *rps12*.

## Discussion

In our study, a total of 21 chloroplast genomes of the genus *Arachis* were retrieved from the NCBI database to explore the applications of their codon usage divergences in the characterizations of several molecular parameters. It was expected that our findings based on a total of 41 genes shared among these chloroplast genomes of the genus *Arachis* would greatly facilitate the studying on molecular characteristics in the genus *Arachis* based on selected genes.

Several molecular parameters, including the codon usage patterns, the ENC values, the RSCU values, the PR2 plot, the neutrality plot, and the GC contents, were calculated for a total of 41 genes shared among a total of 21 chloroplast genomes of the genus *Arachis*. Furthermore, the standard deviations of both the ENC values of each gene and their compositions were calculated to reveal their codon usage divergences (Figs 1D and 2B). The results showed that five genes, including *rps8* (ENC value of 41.413±2.443), *atpF* (43.976±0.623), *ndhE* (46.558±0.659), *ndhG* (50.349±0.683), and *ydf3* (53.625±0.496), showed higher codon usage divergences, while other genes, i.e., *atpB* (ENC value of 47.928±0.049), *ndhF* (42.690±0.089), *psaB* (50.414±0.027), *psbD* (45.887±0.006), *rbcL* (47.370±0.075), *rpoB* (48.100±0.041), *rps2* (46.661±0.096), and *rcf2* (53.269±0.036), showed lower codon usage divergences. These results were consistent with those reported previously, showing broad distributions of ENC values, suggesting that the codon usage bias in chloroplast genomes were influenced by the combined effects of both mutation pressure and natural selection [46]. For example, the gene sequences of *rpl20* were under stronger mutation pressure as suggested by their homogeneous codon usage, while some other genes, e.g., *rps14*, *rps8*, and *petD*, were under both mutation pressure and natural selection pressure as revealed by their uneven and more biased codon usage. To date, the investigations of genetic engineering based on chloroplast genomes are commonly performed [47]; phylogenetic study on some plant suggested typical relationship among chloroplast genomes from different areas [48]. The chloroplast genomes have been established as the ideal markers for both phylogenetic studies [49] and significant contribution to the enhanced resistance to environmental stresses of host plants [50]. It was speculated that our study provided novel insights into the evolutionary characteristics of the genus *Arachis* based on selected genes.

In order to study the base usage diversity in genes of the genus *Arachis* chloroplast genomes, the base usage patterns of 41 genes were evaluated with the mean and the standard deviations of the basic base compositions calculated (Table 1). The base usage diversity of these genes was further assessed by their divergences (Fig 7). The results showed that five genes, i.e., *ndhC* (with the degree of base usage divergence of 0.035), *petD* (0.028), *atpF* (0.026), *rpl14* (0.021), and *rps11* (0.020), were of significantly higher diversity as indicated by their high degrees of base usage divergence. Although the base usage in genes based on the overall compositions was usually considered as the indicator for evaluating the diversity of the gene sequences, this method was not appropriate for evaluating the base mutations [51]. This was because that the diversity of nucleotides in the gene sequences may not lead to any functional changes in the genes. However, the distances among the basic functional units, e.g., the codon usage pattern of sequences determined by the RSCU values, would be more reliable for clustering analysis [52]. Therefore, the RSCU values of the 41 genes were further calculated to explicitly identify the potential variations in their biological functions based on the base mutations in the 21 chloroplast genomes of the genus *Arachis*. Previous studies revealed that the cultivated taxa of *A*. *hypogaea* contained the morphological characters (i.e., runner type habit without floral spikes) similar to those of the wild species of *Arachis* [53]. However, the results in the present study showed that all the chloroplast genomes in the cultivated peanuts (*A*. *hypogaea*) show comparable molecular characteristics (Fig 6). For some organisms, the location of the operon affects the efficiency of protein translation [26,54]. Codon usage characteristics in the genes considered in this paper did not show dependence on their location.

The chloroplast genomes of *Arachia* have been characterized [55,56]. Furthermore, the classification of the genus *Arachis* has also been studied based on the characteristics in the genus *Arachis* chloroplast genomes. For example, the population of *A*. *duranensis* distributed in Salta and Argentina was identified by the combined analysis of chloroplast DNA and non-transcribed spacer 5S rDNA sequences [57]. Our results revealed the narrow genetic base in the cultivated peanuts, which was likely caused by a single polyploidization event isolating the cultivated taxa from the wild species of *Arachis* [58]. Our study identified the evolutionarily conserved characteristics of genes of the genus *Arachis* in their chloroplast genomes to show these general applications in these evolutionary and molecular investigations.

## Conclusions

Advanced molecular techniques have been constantly developed to enhance our understanding of the functions of genes and genomes in peanuts in order to facilitate the studying on the evolution of peanuts based on molecular characteristics. In this study, the patterns of base usage and codon usage based on several molecular characteristics (i.e., the base composition, the ENC, the RSCU, the CBI, and their correlation coefficients) of a total of 41 genes in 21 chloroplast genomes in the genus *Arachis* were investigated to further perform the correspondence and clustering analyses among these genomes. The results revealed significantly higher variations in genomes of wild species than those of the cultivated taxa in the genus *Arachis*, while the codon usage patterns of all 41 genes in the genus *Arachis* were AT-rich with five genes (i.e., *ndhC*, *petD*, *atpF*, *rpl14*, and *rps11*) of higher codon usage divergences, suggesting that the natural selection was the main factor affecting the evolutionary history of these genomes. Furthermore, five genes (i.e., *ndhE*, *ndhG*, *atpF*, *rps8*, and *ycf3*) and nine genes (i.e., *atpB*, *ndhF*, *psbD*, *psaB*, *psbD*, *rbcL*, *rpoB*, *ycf2*, and *rps2*) showed higher and lower base usage divergences, respectively. This study provided novel evidence based on the codon usage patterns to enhance our understanding of the molecular evolution of chloroplast genomes of the genus *Arachis*, facilitating the technical improvement of molecular phylogenetic investigation, and evaluation in the genus *Arachis* based on selected genes.

## Supporting information

S1 File S1 Table Compositional results of 924 concerned sequences. (XLS)
